# Clinicopathological evaluation of the efficacy of endoscopic treatment for sessile serrated lesions comparing endoscopic mucosal resection, cold snare polypectomy, and underwater endoscopic mucosal resection

**DOI:** 10.1002/deo2.70051

**Published:** 2025-01-03

**Authors:** Kaizo Kagemoto, Koichi Okamoto, Yasuyuki Okada, Motoko sei, Shota Fujimoto, Mai Yagi, Takeshi Mitsuhashi, Hiroyuki Ueda, Takanori Yoshimoto, Takanori Kashihara, Tomoyuki Kawaguchi, Yoshifumi Kida, Yasuhiro Mitsui, Yutaka Kawano, Masahiro Sogabe, Hiroshi Miyamoto, Yasushi Sato, Naoki Muguruma, Tetsuji Takayama

**Affiliations:** ^1^ Department of Gastroenterology and Oncology Institute of Biomedical Sciences Tokushima University Graduate School Tokushima Japan; ^2^ Department of Gastroenterology Takamatsu Municipal Hospital Kagawa Japan

**Keywords:** CSP, EMR, inverted growth, SSL, UEMR

## Abstract

**Objectives:**

Recently, various endoscopic treatments for colorectal polyps have been reported, including cold snare polypectomy (CSP) and underwater endoscopic mucosal resection (UEMR), in addition to EMR. However, a precise treatment strategy for sessile serrated lesions (SSL) has not been established. In this study, we analyzed the clinicopathological features of SSL resected by EMR, CSP, and UEMR to determine the most suitable treatment for SSL.

**Methods:**

A total of 92 SSL resected via EMR (*n* = 11), CSP (*n* = 36), and UEMR (*n* = 45) were retrospectively enrolled between February 2021 and October 2022. To evaluate pathological findings, we examined SSL samples, which were stretched before formalin fixation and sectioned at 2‐mm intervals. Primary outcomes were the R0 resection rate and thickness of submucosal (SM) tissue specimens for each treatment. In addition, we evaluated SSL with dysplasia (SSLD) and the inverted growth pattern which may affect the vertical margin.

**Results:**

The R0 resection rate significantly differed among the three groups (EMR, 73%; CSP, 42%; UEMR, 87%, *p* = 0.001). The median thickness of SM tissue resected by CSP (0 µm) was significantly less than that by EMR (362 µm) and UEMR (325 µm; *p* < 0.001). All four SSLDs were diagnosed endoscopically. Five SSLs with inverted growth patterns were pathologically diagnosed. Of these, two SSLs with inverted growth patterns could not be diagnosed endoscopically.

**Conclusions:**

UEMR is considered to be a suitable treatment option for SSL. CSP results were pathologically insufficient. Therefore, surveillance to evaluate local recurrence is important, and the results of further multicenter prospective studies should be referred.

## INTRODUCTION

Colorectal cancer (CRC) is the third most commonly diagnosed cancer, and the second leading cause of cancer‐related death worldwide.^1^ Sessile serrated lesions (SSL) are precursor lesions of the serrated neoplasia pathway, which reportedly causes carcinogenesis in as many as 30% of CRC cases.^2^ The serrated‐neoplasia pathway is associated with BRAF or KRAS mutations and CpG island methylation phenotype, as well as methylation of MLH1 promoter or TP53 mutation.^3^, ^4^, ^5^ Endoscopic treatment is recommended for SSL due to the lesion's malignant potential.^6^ Pathologically, SSL have serrated ducts extending into the crypt base, horizontal growth along the muscularis mucosae (MM), and sometimes inverted growth.^7^, ^8^, ^9^ Therefore, it is necessary to pay attention to the depth of resection during endoscopic treatment.

Endoscopic mucosal resection (EMR) is widely performed for polyp removal.^10^, ^11^ EMR has been performed for SSL, and ESGE guidelines indicate EMR as the preferred treatment for SSL.^11^ On the other hand, cold snare polypectomy (CSP) is a technique that does not require electrocautery.^12^ CSP has become a standard treatment for small nonpedunculated polyps (<10 mm) due to the procedure's efficacy and safety profile.^11^, ^12^, ^13^ Recently, the usefulness of CSP for SSL has been reported.^14^, ^15^ However, due to the characteristics of SSL that extend directly above MM,^16^ there are some concerns about vertical margin in CSP specimens, which often contain no submucosal (SM) tissue.^17^, ^18^ Underwater EMR (UEMR), which has emerged as an alternative EMR technique, is easily performed without submucosal injection.^19^ Recently, UEMR has been reported to be effective for removing flat or large colorectal lesions.^20^ Therefore, UEMR is expected to be a suitable technique for SSL; however, there have been few reports about UEMR for SSL treatment, especially with regard to the analysis of vertical margins, such as submucosal resection depth.

Thus, a variety of endoscopic treatments for colorectal lesions have been reported, although a precise treatment strategy for SSL has not been established. In order to determine the most suitable treatment for SSL, it is necessary to evaluate the vertical margin and resection depth for each treatment, due to the characteristics of SSL.^16^ Therefore, in the present study, we retrospectively analyzed the R0 resection rate and thickness of SM tissue of SSL specimens resected by EMR, CSP, and UEMR.

## METHODS

### Patients and study design

This was a single‐center retrospective study designed to evaluate endoscopic treatment for SSL. We enrolled consecutive patients who underwent endoscopic treatment for SSL at Tokushima University Hospital between February 2021 and October 2022. To accurately evaluate pathological findings, stretched specimens before formalin fixation were examined. Lesions that could not be diagnosed with high pathological confidence, due to factors such as fragmentation, were excluded. All lesions were resected with EMR, CSP, or UEMR. Patients receiving an antithrombotic drug were incorporated into the trial. The study protocol was approved by the ethics committee of Tokushima University Hospital. The trial was registered in the University Hospital Medical Information Network Clinical Trials Registry (UMIN000049966). Written informed consent was obtained from patients prior to study participation.

### Procedure

All procedures were carried out by experienced endoscopists (at least 1000 colonoscopies) with a high‐definition video colonoscope (PCF‐H290Z [Olympus] and EC‐L600ZP7 [Fujifilm]). A total colonoscopy was performed with CO_2_ insufflation. When the target lesion was detected, it was diagnosed by narrow‐band imaging (NBI) or blue laser imaging (BLI) in addition to white light imaging (WLI), optionally with chromoendoscopy. All lesions were measured by an opened snare, and morphology was classified according to the Paris classification.^21^ Subsequently, endoscopists selected a treatment method based on the endoscopic appearance and size of the lesions. If the SSL without dysplasia was less than 10 mm, CSP was performed; if it was greater, EMR or UEMR was performed. EMR procedures were performed as previously described.^10^ First, normal saline was injected locally into the submucosa of SSL, then the lesion was captured by snare and resected with an electrocautery device (Endo‐cut Q, Effect2, Duration1, and Interval4; VIO300D; ERBE). CSP procedures were performed without submucosal injection and electrocautery.^13^ UEMR procedures were performed according to previous reports as follows^19^: deflation of the colorectal lumen; immersion of water until complete filling of the lumen using mechanical water pump (OFP‐2; Olympus); capture and resection of the lesion by snare with electrocautery (same setting as EMR). The polypectomy snares used were chosen by endoscopists depending on the situation (Snaremaster, Snaremaster Plus; Olympus). Closure of the wound with a clip was performed at the discretion of the operator. Antithrombotic drugs were continued or discontinued based on Japanese gastrointestinal endoscopy guidelines for patients undergoing antithrombotic drugs.^22^, ^23^

### Pathological examination

All resected specimens were fixed in 10% formalin with stretching, sectioned at 2‐mm intervals, and stained with hematoxylin and eosin. CSP specimens often cannot be evaluated accurately, because of retrieval failure or fragmentation.^24^, ^25^ For this reason, we used stretched specimens prior to formalin fixation. This would allow us to accurately evaluate the differences in pathological features between treatments. Pathological examinations were performed by pathologists who were board‐certified by the Japanese Society of Pathology. SSL including SSL with dysplasia (SSLD) was diagnosed according to the World Health Organization's 2019 classification system. Inverted growth type SSL was diagnosed with the findings of crypts with the invagination into the submucosal layer.^7^, ^8^ The endoscopists and pathologists were consulted to assess whether the specimens contained MM and submucosal tissue.

### Evaluated parameters

The primary outcomes were the R0 resection rate and the thickness of SM tissue of specimens for each treatment method. R0 resection included horizontal margin (HM) 0 and vertical margin (VM) 0. The resected specimen was defined as VM0 when the MM was confirmed, when the MM was fragmented or disappeared as undeterminable (VMX), and when the gland ducts could not be confirmed to the bottom as VM1. The thickness of SM tissue that was measured from the MM to the vertical resection margin of submucosal tissue was evaluated at the center (a) and the average of both edges (b) of specimens. Secondary outcomes included tumor diameter, complications, SSLD, and the inverted growth pattern which may affect the VM. Patients were evaluated for complications of delayed bleeding and perforation.

### Statistics

The chi‐square test was used to compare categorical outcomes. Kruskal‐Wallis test and Dunn's multiple comparisons test were used to compare continuous outcomes. *p*‐Values < 0.05 were considered statistically significant.

## RESULTS

### Participant flow

**FIGURE 1:**
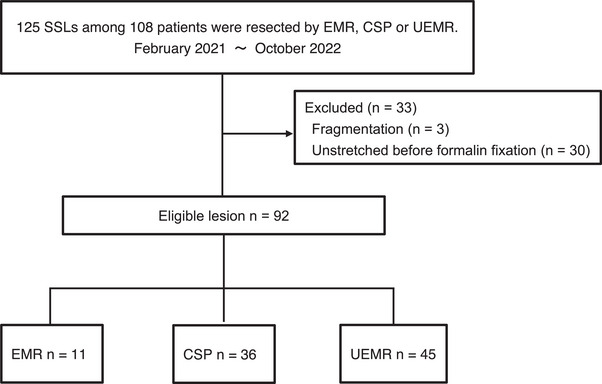
Flowchart of the study. EMR, endoscopic mucosal resection; CSP, cold snare polypectomy; UEMR, underwater endoscopic mucosal resection.

Between February 2021 and October 2022, 108 patients with 125 SSLs underwent endoscopic resection by EMR, CSP, or UEMR. To evaluate precise pathological findings, we enrolled lesions that were stretched before formalin fixation and sectioned at 2‐mm intervals. We excluded 33 lesions that were fragmented (*n* = 3) or unstretched before formalin fixation (*n* = 30; EMR 8, CSP 8, UEMR 14). Thus, we enrolled 92 SSLs in this study. Of these, 11 lesions were resected by EMR, 36 by CSP, and 45 by UEMR (Figure 1).

### Clinicopathological characteristics

Table 1 shows the clinicopathological characteristics of patients and lesions. There were no significant differences in age, gender, location, and morphology between the three groups. Most of the lesions were located at the proximal colon and were type 0‐IIa morphologically. The lesion size of CSP tended to be smaller than that of EMR and UEMR, and there was a significant difference (*p* < 0.01). A total of 19 serrated polyposis syndrome (SPS) cases were observed, of which two were EMR, six were CSP, and 11 were UEMR. There were four SSLDs overall: one lesion in the EMR group and three lesions in the UEMR group. A total of five SSLs with inverted growth patterns were detected, including one lesion in the EMR group, one lesion in the CSP group, and three lesions in the UEMR group.

**TABLE 1:** Baseline characteristics of the study subjects.

	ALL (*n* = 92)	EMR (*n* = 11)	CSP (*n* = 36)	UEMR (*n* = 45)	*p*‐value
Male/female, *n*	49/43	6/5	19/17	24/21	0.98
Median age, years (range)	65 (48–88)	63 (52–81)	65 (49–88)	65.5 (48–82)	0.75
Location, *n* (%) proximal	81 (88%)	9 (82%)	33 (92%)	39 (87%)	0.67
distal	10 (12%)	2 (8%)	3 (8%)	6 (15%)	
Median size, mm (range)	10 (3–35)	10 (4–35)	8 (3–12)	12 (4–35)	<0.01
Morphology, *n* (%) Is	3 (3%)	0 (0%)	1 (3%)	2 (4%)	0.91
IIa	89 (97%)	11 (100%)	35 (97%)	43 (36%)	
SPS	19 (20%)	2 (18%)	6 (19%)	11 (24%)	0.77
SSLD	4 (4%)	1 (9%)	0 (0%)	3 (7%)	0.23
SSL with the **i**nverted growth pattern	5 (5%)	1 (9%)	1 (3%)	3 (7%)	0.54

Abbreviations: CSP, cold snare polypectomy; EMR, endoscopic mucosal resection; SPS, serrated polyposis syndrome; SSL, sessile serrated lesions; SSLD, sessile serrated lesions with dysplasia; UEMR, underwater endoscopic mucosal resection.

### Therapeutic outcomes

**FIGURE 2:**
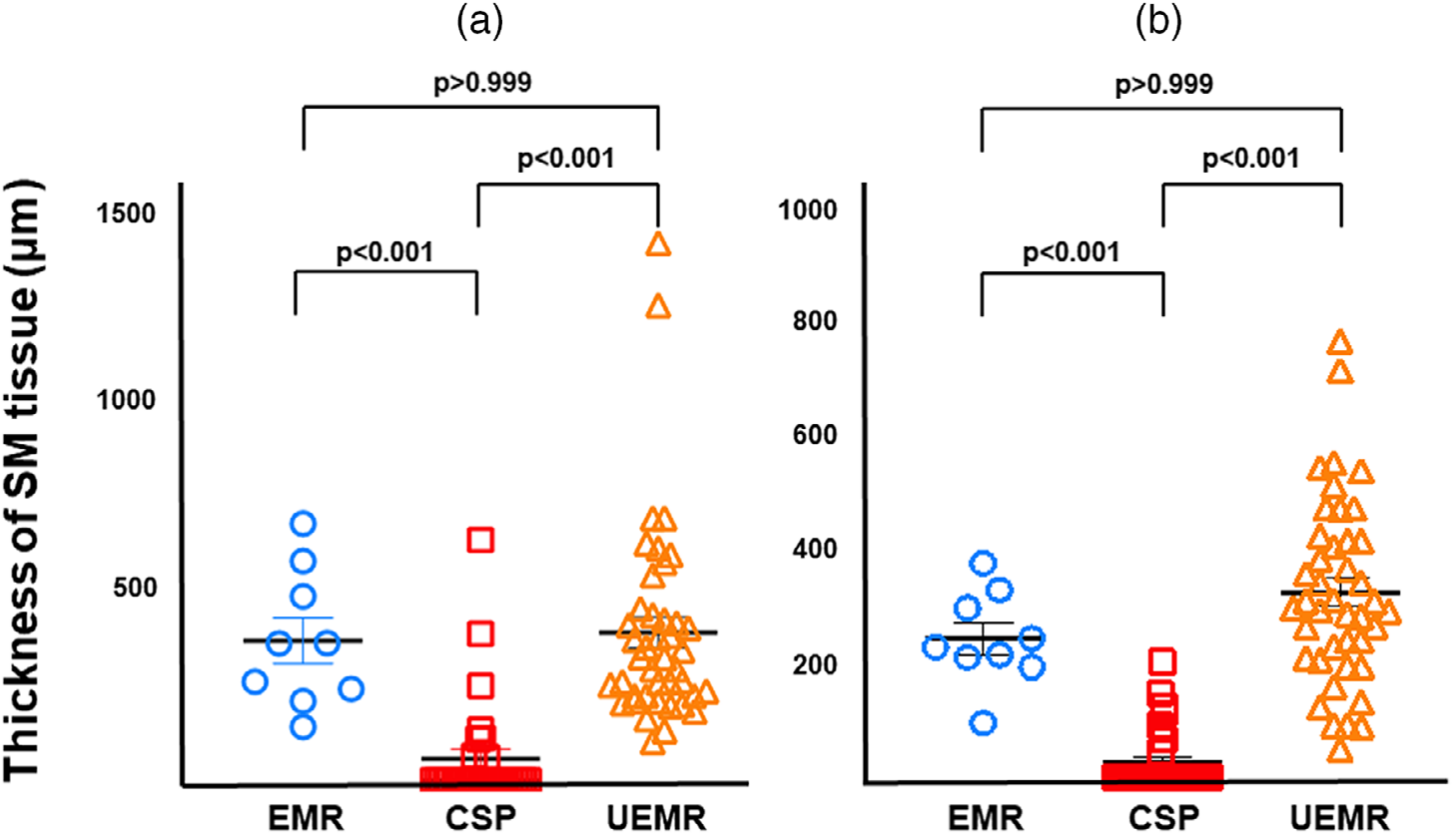
Thickness of submucosal (SM) tissue in specimens among the three groups. The parameters were evaluated at the central part (a) and the average of both edges (b) of resected specimens. (a) The median thickness of SM tissue at the center of specimens resected by cold snare polypectomy (CSP; 0 µm) was significantly less than that by endoscopic mucosal resection (EMR; 362 µm) and underwater EMR (UEMR; 325 µm), but there was no significant difference between the two groups. (b) The median thickness of SM tissue at the average of both edges resected by CSP (0 µm) was also significantly less than that by EMR (237 µm) and UEMR (303 µm), but there was no significant difference between the two groups.

**FIGURE 3:**
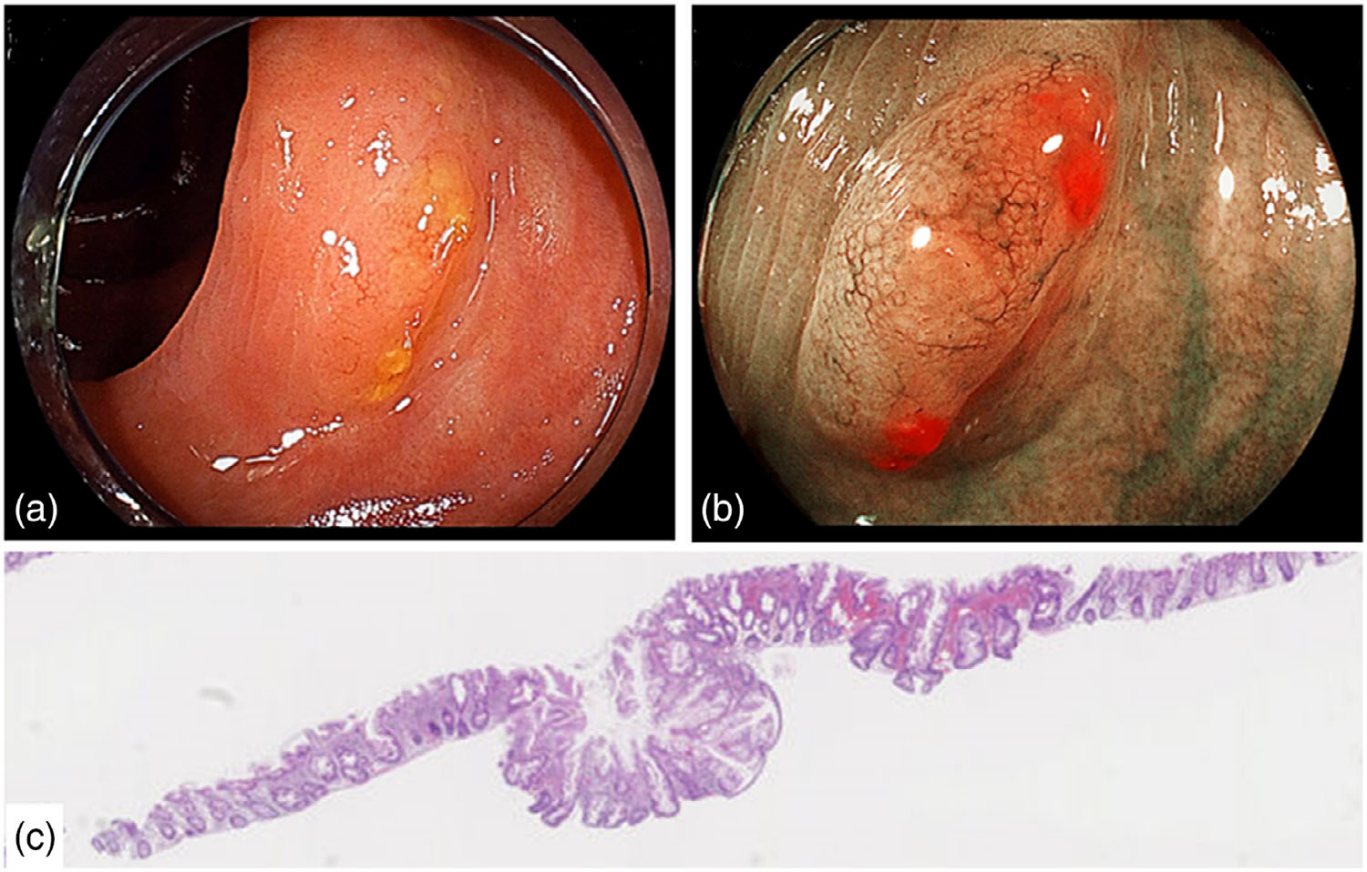
A case of sessile serrated lesions with inverted growth pattern resected by cold snare polypectomy. (a) The slightly elevated lesion was 6 mm in diameter and located in the transverse colon. (b) Expanded crypt opening and thick and branched vessels are observed using magnifying blue laser imaging. This lesion had two components and was relatively well‐demarcated. Near the center of the lesion, there was an area of indistinct glandular ducts and dilated inter‐glandular spaces. (c) Pathologically, crypts were invaginated into deeper layers in an area with unclear surface structure. Serrated ducts extending into the crypt base were seen but muscularis mucosae had disappeared.

**FIGURE 4:**
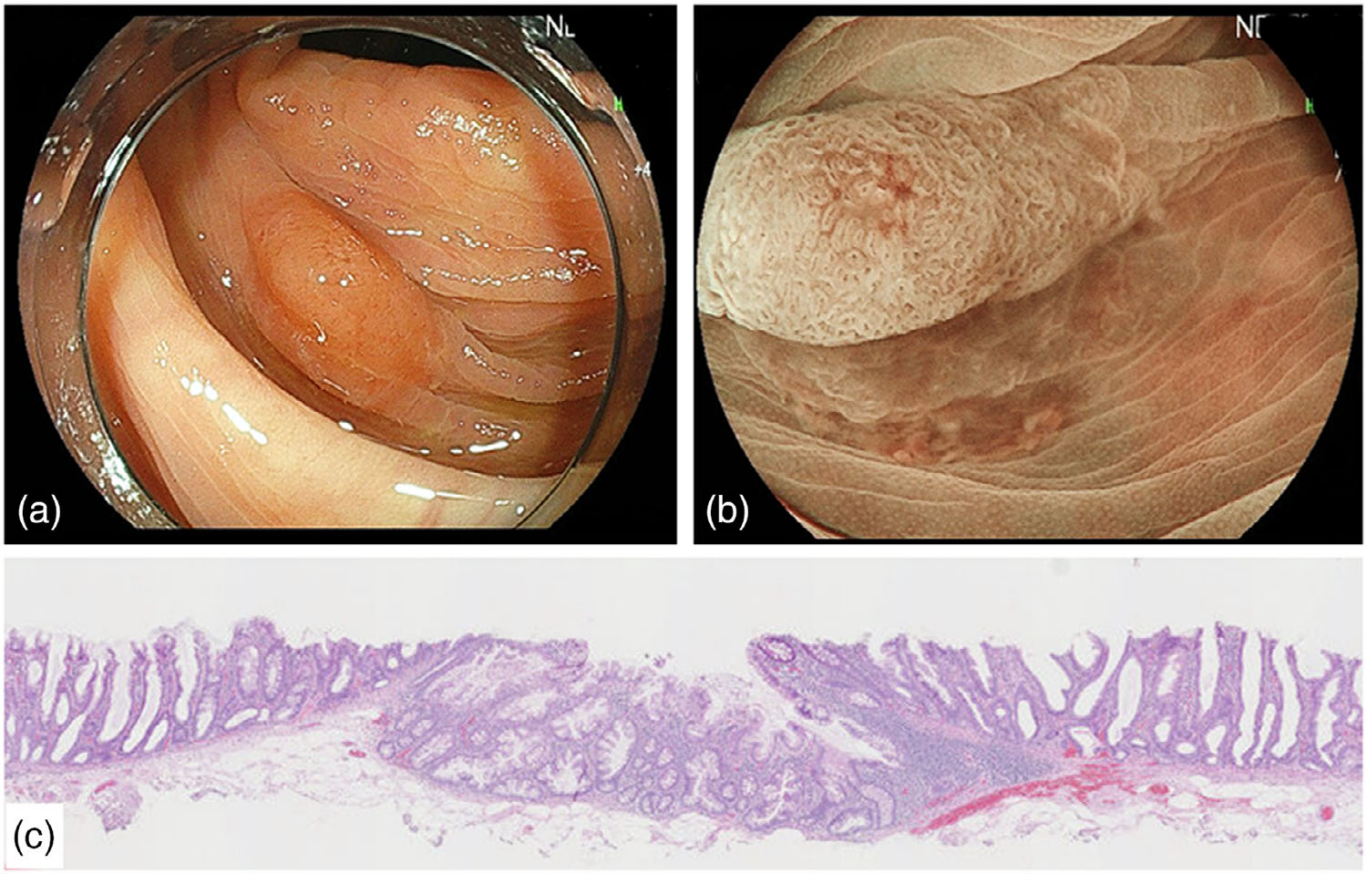
A case of sessile serrated lesions with inverted growth pattern resected by underwater endoscopic mucosal resection. (a) The slightly elevated lesion was 12 mm in diameter and located in the transverse colon. (b) Expanded crypt openings are observed using underwater magnifying blue laser imaging. Near the center of the lesion, there was a boundary indistinct area with lower glandular duct density and dilated inter‐glandular spaces. (c) Pathologically, inverted growth was observed in an area with unclear surface structure. Serrated ducts extending into the crypt base were seen and muscularis mucosae was confirmed.

Table 2 shows the procedure‐related outcomes. The R0 resection rate significantly differed among the three groups (EMR 73%; CSP 42%; UEMR 87%, *p* = 0.001). VM0 resection was achieved in 10 cases with EMR (91%), 16 cases with CSP (44%), and 42 cases with UEMR (93%), and there was a significant difference (*p* < 0.001). There were one VMX and no VM1 cases in the EMR group, 18 VMX and two VM1 cases in the CSP group, and three VMX and no VM1 cases in the UEMR group. In terms of HM0 resection, there was no significant difference (EMR 73%; CSP 70%; UEMR 89%, *p* = 0.12). There were three HMX and no HM1 cases in the EMR group, 10 HMX and one HM1 cases in the CSP group, and five HMX and no HM1 cases in the UEMR group. Complications such as delayed bleeding and perforation were not observed. The median thickness of SM tissue at the center of specimens resected by CSP (0 µm) was significantly less than that by EMR (362 µm) and UEMR (325 µm; *p* < 0.001), but there was no significant difference between the two groups (*p* > 0.999). The median thickness of SM tissue at the average of both edges resected by CSP (0 µm) was also significantly less than that by EMR (237 µm) and UEMR (303 µm; *p* < 0.001), but there was no significant difference between the two groups (*p* > 0.999; Figure 2). The piecemeal resection was performed in one case of EMR and two cases of UEMR, both of which were reconstructed and allowed for an accurate pathological diagnosis. In this study, all four SSLD cases received R0 resection by EMR and UEMR. Five SSL cases with inverted growth patterns were pathologically diagnosed. Of these, the one lesion resected by CSP was VMX (Figure 3). On the other hand, the four lesions resected by EMR and UEMR were VM0 (Figure 4). Two SSLs with inverted growth patterns (Figures 3 and 4) could not be diagnosed endoscopically.

**TABLE 2:** Procedure‐related outcomes of the three groups.

	EMR (*n* = 11)	CSP (*n* = 36)	UEMR (*n* = 45)	*p*‐value
R0 resection, *n* Rate (%)	8 73%	15 42%	39 87%	0.001
VM 0, *n* (%)	10 (91%)	16 (44%)	42 (93%)	<0.001
VM X/1, *n*	1 / 0	18 / 2	3 / 0	
HM 0, *n* (%)	8 (73%)	25 (70%)	40 (89%)	0.12
HM X/1, *n*	3 / 0	10 / 1	5 / 0	
Complications				
Delayed bleeding	0	0	0	
Perforation	0	0	0	

Abbreviations: CSP, cold snare polypectomy; EMR, endoscopic mucosal resection; HM, horizontal margin; UEMR, underwater endoscopic mucosal resection; VM, vertical margin.

## DISCUSSION

In this study, we evaluated the outcomes of endoscopic treatment for SSL. UEMR demonstrated an excellent R0 resection rate (EMR 73%; CSP 42%; UEMR 87%, *p* = 0.001). In addition, the SM tissue of specimens resected by UEMR was well contained more than with CSP and not different from EMR. Furthermore, UEMR achieved R0 resection for all 3 SSLD lesions. Therefore, UEMR is considered to be a suitable treatment option for SSL and SSLD. On the other hand, CSP results were pathologically insufficient because of the lower R0 resection rate.

CSP had a particularly low VM0 resection rate, and the SSL with an inverted growth pattern was also treated by VMX resection. Therefore, we concluded that the indication of CSP for SSL should be determined carefully, although it seemed to be important to investigate the local recurrence of the lesion. There are few reports that have evaluated endoscopic treatment for SSL based on precise pathological results, and no published studies to date have investigated inverted growth‐type SSL treated by CSP. High confidence can be placed in our results because, in the present study, we precisely evaluated SSL specimens that were stretched and sectioned at 2‐mm intervals.

Conventional EMR is an established method for removing advanced colorectal polyps.^10^, ^11^ In the present study, EMR demonstrated an R0 resection rate of 73% (VM 91%; HM 73%), which seemed better compared with previous reports.^20^, ^26^ There were three cases of RX resection in the EMR group, all of which were HMX, and one case of VMX. Therefore, HMX was thought to be the main cause of RX resection. UEMR demonstrated better performance as indicated by an R0 resection rate of 87% (VM 93%; HM 89%). There were six cases of RX resection in the UEMR group, of which five cases were HMX. HMX was also the main cause of RX resection in the UEMR group. The difference in R0 resection rates between EMR and UEMR was mainly due to the difference in HM. Previous reports have shown that UEMR is superior to EMR in en bloc resection and R0 resection, consistent with our data.^20^, ^27^ The underwater technique improves endoscopic visibility and facilitates the evaluation of the borders of lesions. In addition, water immersion decreases the luminal extension force and, subsequently, sessile or flat lesions become polypoid, which can then be easily captured by snare.^19^ In contrast, submucosal injection often enlarges the lesion and makes snare capture more difficult.^28^, ^29^ Particularly, the effects may be a major disadvantage with SSL resection, which is thought to have contributed to our results. In addition, since UEMR does not require submucosal injection, we confirmed the resection depth and found that the SM tissue was sufficiently contained in the resected specimens, as reported by Toyosawa et al., and there was no significant difference with EMR.^30^ UEMR was also considered useful for the resection of SSL.

On the other hand, CSP demonstrated a lower R0 resection rate of 42% (VM 44%; HM 70%) and VM was more likely to be the cause of RX resection than HM. In addition, as in previous reports, CSP specimens contained little SM tissue and the median thickness of SM tissue was 0 µm.^18^, ^30^ Thus, shallow resection depth of CSP was considered to be the main cause of inadequate VM. It has been previously reported that SSL is the risk factor associated with incomplete resection in CSP, because of the difficulty of recognizing the lesion's border.^31^ In our study, we used high‐definition video colonoscopy in combination with NBI, BLI, or chromoendoscopy to observe the border. As a result, we were able to better recognize the boundaries of lesions, resulting in an HM0 of 70% in CSP, with no significant difference between the three groups. Our data seem to support the reports by Kimoto et al. and Hatten et al. demonstrating the usefulness of CSP for SSL and low recurrence rate.^14^, ^15^ Compared with these previous reports, VM resected by CSP was pathologically insufficient in the present study, but it may not significantly contribute to clinical outcomes such as recurrence. To confirm this possibility, the local recurrence rate of those lesions should be assessed. In this study, all SSLD could be differentiated endoscopically, and R0 resection was achieved by EMR or UEMR. Previous reports have demonstrated the high performance of endoscopic diagnosis for SSLD, which has features of an adenomatous pattern such as pit patterns of type III or IV according to Kudo's classification and Type 2A, 2B, or 3 of the Japan NBI Expert Team (JNET) classification.^32^, ^33^ In the present study, it was possible to differentiate SSLD endoscopically consistent with previous reports. On the other hand, there were cases in which SSL with inverted growth patterns were difficult to differentiate endoscopically. The endoscopic feature of SSL with an inverted growth pattern was reported to have a depression area in the center of the lesion.^8^, ^9^ In the present study, three of the five SSLs with inverted growth patterns could be diagnosed endoscopically based on the depressed area, as previously reported. Conversely, two lesions had only slight findings as shown in Figures 3 and 4, and could not be diagnosed. Comparing the characteristics of SSL with inverted growth pattern, which is invagination in the submucosa and the shallow resection depth of CSP, these lesions may become a risk factor for incomplete resection of VM and local recurrence of SSL resected by CSP. Therefore, careful surveillance might be required for these lesions resected by CSP more closely. Recently, the indication of CSP for SSL has been gradually expanding.^34^ CSP may be a reasonable treatment option for SSLs less than 20 mm, as they have a low risk of SSLD or invasive cancer and can be endoscopically differentiated.^15^, ^35^ However, the CSP for SSL was pathologically insufficient. These results suggest that careful observation after CSP is important, and results of further multicenter prospective studies should be referred to.

Our study has several limitations. First, it was performed at a single institute with a small number of cases. Second, our study utilized a retrospective design. Lesion size was significantly different for each technique and the sample size of EMR was smaller because the procedure was chosen based on the endoscopic appearance of the lesions. In addition, this study included the stretched specimens. The fragmented or unstretched lesions were excluded and, therefore, selection bias cannot be excluded.

In conclusion, we evaluated precise pathological outcomes of endoscopic treatment for SSL. UEMR is considered to be a suitable treatment option for SSL pathologically. In addition, UEMR is expected to be adapted to SSLD. On the other hand, CSP results were pathologically insufficient, although the usefulness of CSP for SSL without dysplasia has been clinically demonstrated. It may be desirable to determine treatment options for SSL based on size and surface structure, and further clinical trials should be referred.

## CONFLICT OF INTEREST STATEMENT

Tetsuji Takayama has received grants from Fujifilm. The other authors declare no conflict of interest.

## ETHICS STATEMENT

**Approval of the research protocol by an Institutional Reviewer Board**. The study protocol was approved by the ethics committee of Tokushima University Hospital.

## PATIENT CONSENT STATEMENT

Informed consent was obtained from patients prior to study participation.

## CLINICAL TRIAL REGISTRATION

The trial was registered in the University Hospital Medical Information Network Clinical Trials Registry (UMIN000049966)
